# Neoadjuvant tislelizumab and tegafur/gimeracil/octeracil (S-1) plus oxaliplatin in patients with locally advanced gastric or gastroesophageal junction cancer: Early results of a phase 2, single-arm trial

**DOI:** 10.3389/fonc.2022.959295

**Published:** 2022-08-30

**Authors:** Yuping Yin, Yao Lin, Ming Yang, Jianbo Lv, Jiaying Liu, Ke Wu, Ke Liu, Anshu Li, Xiaoming Shuai, Kailin Cai, Zheng Wang, Guobin Wang, Jianfeng Shen, Peng Zhang, Kaixiong Tao

**Affiliations:** ^1^Department of Gastrointestinal Surgery, Union Hospital Tongji Medical College Huazhong University of Science and Technology, Wuhan, China; ^2^Department of Pathology, Union Hospital, Tongji Medical College, Huazhong University of Science and Technology, Wuhan, China; ^3^Department of Ophthalmology, Ninth People’s Hospital, Shanghai JiaoTong University School of Medicine, Shanghai, China

**Keywords:** tislelizumab, PD-L1 inhibitor, combination therapy, neoadjuvant therapy, gastric cancer, machine learning algorithm

## Abstract

**Background:**

Recently, the combination of immunotherapy with chemotherapy has been recommended as first-line treatment of metastatic gastric/gastroesophageal junction (G/GEJ) in the clinical guidelines of many countries; the therapeutic potential of this application needs to be further investigated for neoadjuvant therapy of advanced G/GEJ cancer patients.

**Methods:**

We performed a prospective, single-arm, open-label, phase 2 trial of the PD-1 inhibitor tislelizumab combined with S-1 plus oxaliplatin (SOX) in patients with advanced LAG/GEJ cancer. All patients underwent the three-cycle (21 days/cycle) treatment except for one patient who underwent two cycles. The primary endpoints were tumor major pathology response (MPR) and other events of tumor response assessed by the RECIST 1.1 and Becker criteria. Moreover, we constructed a few-shot learning model to predict the probability of MPR, which could screen those patients who might benefit from the neoadjuvant immunotherapy–chemotherapy scheme. This study was registered at https://clinicaltrials.gov/ct2/show/NCT0-4890392.

**Results:**

Thirty-two patients were enrolled; 17 patients (53.1%) achieved MPR (≤10% viable tumor cells) after treatment, and among them, 8 (25.0%) had a pathological complete response (pCR). The 1-year overall survival (OS) rate was 91.4% and the 1-year recurrence-free survival (RFS) rate was 90.0%. Adverse events occurred in 24 patients (65.6%) and grade III–IV adverse events were observed in 4 patients (12.5%) during the neoadjuvant period. Furthermore, we found commonly used preoperative assessment tools such as CT and EUS, which presented limited accuracy of tumor therapeutic response in this study; thus, we developed a therapeutic response predictive model that consisted of TNFα, IFNγ, IL-10, CD4, and age of patient, and the AUC of this FSL model was 0.856 (95% CI: 0.823–0.884).

**Discussion:**

Our study showed that the neoadjuvant PD-1 inhibitor tislelizumab combined with SOX had promising application potential and presented no increasing treatment-related adverse events in patients with advanced G/GEJ cancer. Moreover, the predictive model could help therapists to evaluate the therapeutic response of this scheme accurately.

**Clinical Trial Registration:**

https://clinicaltrials.gov/ct2/show/NCT0-4890392, identifier [NCT04890392].

## 1 Introduction

Gastric/gastroesophageal junction (G/GEJ) cancer is the fifth most common cancer and the third leading cause of cancer mortality worldwide (1, 2). There were 1,033,701 new cases of gastric cancer (representing 5.7% of overall cases) and 782,685 deaths related to gastric cancer in 2018, about half of which lived in East Asian countries, which had the highest mortality rate (3). Although the European Society for Medical Oncology (ESMO) and National Comprehensive Cancer Network (NCCN) clinical practice guidelines recommended neoadjuvant treatment for patients with G/GEJ cancer in the T2–4 stage, this strategy was not given enough importance in eastern countries in the past decades.

Immuno-oncologic agents targeting the programmed death-1 (PD-1)/programmed death-ligand 1 (PD-L1) axis have shown promising anti-cancer effects in several malignant diseases. PD-L1 binding with PD-1, an immunoinhibitory receptor expressed on T cells, thus suppresses T-cell-mediated immune responses (4). Importantly, a previous study (5) reported that the prognosis was poorer in GC patients with higher PD-L1 expression than those patients with lower PD-L1 expression, although immune checkpoint blockade (ICB) drugs such as nivolumab have been reported to be effective in patients with unresectable, advanced, or recurrent G/GEJ cancer, which was validated by preclinical findings (6–8). Recently, due to the inspiring results of KEYNOTE-062 and CheckMate-649 clinical trials (8, 9), the therapeutic value of immunotherapy combined with chemotherapy was validated in unresectable advanced G/GEJ cancer, which gives us rational reasons to further test the application of this strategy in neoadjuvant therapy of advanced G/GEJ cancer patients.

For this, we conducted a single-arm, phase 2 trial of the PD-1 inhibitor tislelizumab combined with S-1 plus oxaliplatin to evaluate the neoadjuvant therapeutic effect in patients with advanced G/GEJ cancer. Moreover, the predictive biomarkers of immunotherapy response are under investigation, and traditional biomarkers such as PD-L1 CPS score, TMB, and MSI-H are widely known, but a previous study reported that these biomarkers had some limitations in predicting the response of chemotherapy combined with immunotherapy in cancer treatment. Moreover, immune cytokines such as TNF-α, INF-α, and IL-10 in the tumor microenvironment are involved in the immune reaction; thus, we monitored a variety of these cytokines in neoadjuvant therapy, by which we established a predictive model to screen out patients who might benefit from the neoadjuvant immunotherapy–chemotherapy formula.

## 2 Materials and methods

### 2.1 Study design

A single-arm, open-label, phase 2 trial (NCT04890392) of the PD-1 inhibitor tislelizumab combined with S-1 plus oxaliplatin (SOX) was performed in patients diagnosed with locally advanced G/GEJ cancer from September 2020 to March 2021. All patients received treatment in the Department of Gastrointestinal Surgery, Union Hospital, Tongji Medical College, Huazhong University of Science and Technology. Treatment was performed for three cycles (21 days/cycle) and discontinued in case of disease progression, unacceptable toxicity, or consent withdrawal. The major pathology response (MPR) rates were considered as primary endpoints and secondary endpoints were identified as overall disease control rates, pathological complete response (pCR) rates, adverse events (AEs), 1-year overall survival (OS), and recurrence-free survival (RFS) in this study.

Patients were evaluated for tumor pathology regression after D2 radical surgery, and were followed up. The study was approved by the institutional review boards at all sites, and conformed to the Declaration of Helsinki guidelines. All patients provided written informed consent.

### 2.2 Inclusion and exclusion criteria of patients

Briefly, patients with locally advanced HER2-negative G/GEJ cancer, Eastern Cooperative Oncology Group performance status of 0 or 1, and no prior chemotherapy except neoadjuvant or adjuvant chemotherapy completed ≥180 days before randomization were included. Additional details were provided as supplementary material (**Supplementary Table S1**).

### 2.3 Treatment

Patients received the neoadjuvant tislelizumab (200 mg intravenously once in 3 weeks) plus the SOX scheme (S-1, 40 mg/m^2^ orally twice daily for 14 days followed by 7 days off; oxaliplatin, 130 mg/m^2^ intravenously on day 1 every 3 weeks) and received D2 radical surgery.

Patients were treated with the postoperative adjuvant SOX scheme for five cycles, S-1, 40 mg/m^2^ orally twice daily for 14 days followed by 7 days off; oxaliplatin, 130 mg/m^2^ intravenously on day 1 every 3 weeks after D2 radical surgery. Additional details are provided as supplementary material (**Supplementary Table S2**).

### 2.4 Clinical data collection and assessment

All patient data were recorded by the physician-in-charge with a certificate of clinical trial, and extracted from the electronic medical record system. Demographic data included sex, age, and Eastern Cooperative Oncology Group (ECOG) score (10). Pathological indicators included tumor location, maximal diameter of the tumor (cm), depth of tumor invasion before treatment (cm), Borrmann type, clinical TNM Classification of Malignant Tumors (cTNM) of Eighth Edition Gastric Cancer Staging of American Joint Committee on Cancer (AJCC) (11, 12) before treatment, history of the tumor tissue, nerve invasion, and vascular invasion.

We also included baseline laboratory indicators such as white blood cell (WBC) count, red blood cell (RBC) count, and blood platelet (PLT), hemoglobin (Hb), glutamic oxaloacetic transaminase (AST), and alanine transaminase (ALT) levels. Hematological inflammation biomarkers were assessed as potential valuable factors. Hence, we included platelet–lymphocyte ratio (PLR), neutrophil–lymphocyte ratio (NLR), and prognostic nutritional index (PNI), and the PNI was calculated as 10 × serum albumin (g/dl) + 0·005 × lymphocyte count (per mm^3^). Likewise, immunological indicators such as CD3+, CD4+, and CD8+ cell amounts, as well as IL-2, IL-4, IL-6, IL-10, tumor necrosis factor (TNF)-α, and IFN-γ levels were also detected in peripheral blood collected from patients before treatment and each time after cycle.

### 2.5 Safety assessment

During the treatment, two physicians with intermediate professional qualification used the National Cancer Institute Common Terminology Criteria for Adverse Events (version 5.0) (13) to evaluate and record AEs, including myelosuppression, leukopenia, neutrophil count decrease, platelet count decrease, hypoproteinemia, wound complications, abdominal infection, pulmonary infection, pleural effusion, pulmonary embolism, dermatitis, thrombotic events, gastroparesis, anemia, postoperative bleeding, anastomotic leakage, ileus, vomiting, diarrhea, constipation, abdominal distention, icterus, acute renal insufficiency (ARI), hypohepatia, gamma-glutamyl transferase increase, aspartate aminotransferase increase, and alanine aminotransferase increase in preoperative, postoperative, and adjuvant therapy schedules. One physician with senior professional qualification verified and finalized these records.

### 2.6 Tumor regression assessment

Tumors were evaluated according to Response Evaluation Criteria in Solid Tumors (RECIST) v1.1 based on contrast-enhanced CT and positron emission computed tomography (PET) Response Criteria in Solid Tumors (PERCIST) at baseline and before surgery. Tumor staging was also performed at baseline (cTNM) and after surgery according to Eighth Edition Gastric Cancer Staging of AJCC. Contrast-enhanced CT was performed before the first dose of adjuvant treatment and every 3 months until disease relapse or death, for up to 1 year after surgery. Pathological response of the primary tumor after surgery was graded according to Becker criteria of Tumor Regression Grade (TRG) (14). Primary lesions and tumor volume were assessed by physical and pathological measurements before neoadjuvant immunotherapy–chemotherapy treatment. Imageological examinations (three-dimensional abdominal CT) were confirmed each time after cycle of treatment. Pathologic response in the primary lesion was assessed after D2 radical surgery by a pathologist. TRG was applied in assessment and TRG 1a = no residual tumor cells; TRG 1b = single cells or small groups of cells (<10%, residual tumor cells); TRG 2 = residual cancer with desmoplastic response (10%–50%, residual tumor cells); and TRG 3 = minimal evidence of tumor response (>50%, residual tumor cells) (15). The MPR was defined as TRG = 1a or 1b.

### 2.7 Prognosis assessment

Regular medical follow-up data were obtained using telephone calls, clinic visits, internet, and other interaction tools. The OS was defined as the duration from the first time diagnosed as having LAG/GEJ cancer to the earliest evidence of death or the end of follow-up, and RFS was defined as the duration from the first time diagnosed as having LAG/GEJ cancer to the earliest evidence of recurrence or the end of follow-up. Patients were followed up until 20 May 2022.

### 2.8 Predictive model construction by few-shot learning

Few-shot learning (FSL) was more suitable to help classify high-dimensional cytokine data from a small sample size (16). To better predict MPR, we constructed a filtering algorithm to rank the features and selected the most efficient model from seven FSL machine-learning classifiers (17): *k*-nearest neighbor (classif.kknn), random forest (classif.ranger), Gaussian processes (classif.gausspr), Naive Bayes processes (classif.naive_bayes), factorization machine supported neural network (classif.fnn), support vector machines (classif.svm), and logistic regression (classif.log_reg). After filtering demographic data, clinicopathological data, baseline laboratory indicators, and immunological indexes, and applying those FSL models, we resampled results by *k*-fold (*k* = 1, 3, 5) cross-validation method (18) and calculated results of the receiver operating characteristic curve (ROC) analysis and classification errors (classif.ce) (19).


$$classif.ce=\frac{1}{n}\sum_{i=1}^{n}w_{i}\left(t_{i}\neq r_{i}\right)$$

Then, we randomly constructed a training set including all randomly selected samples with different scales (*n* = 18, 20, 22, 24, and 26) of overall data and a test set that included all patients (*n* = 32) to develop the predictive model.

### 2.9 Statistical analysis

A total of 25 patients treated with neoadjuvant therapy would provide 80% power to detect an MPR rate of 50% at a one-sided 2.5% alpha level under the null hypothesis of the MPR equal to 40%. Considering a 10% discontinuation rate, 32 assessable patients were enrolled in the study. Continuous variables were expressed as mean ± standard deviation (σ) and interquartile range (IQR), while categorical variables were reported using absolute frequency and percentage. Descriptive comparisons were made by the Pearson’s *χ*^2^ test for categorical variables and the Mann–Whitney *U* rank sum test for continuous variables. For filtering features, we calculated the property “importance” with integrated feature selection methods to determine significant predictors. Next, we applied benchmark experiments to compare FSL models by the R “mlr3” package (19). After that, the predictive model was established through a train set and a test set. To validate the model, the area under the ROC curve (AUC) was determined. Furthermore, to interpret the behavior and explain predictions of the FSL machine learning model, we explained the features’ importance on the results of the predictive model and draw partial dependence plots (20) (PDP) of features to show the marginal effect on the predicted outcome by the R “iml” package (21).

The SPSS software version 24·0 was used for statistical analysis, and R 4.0.1 and Prism version 8.0 were used for analyzing and mapping results. A bilateral *p* < 0.05 was considered statistically significant.

## 3. Results

### 3.1 Demographics and baseline clinicopathological characteristics

**Figure 1:**
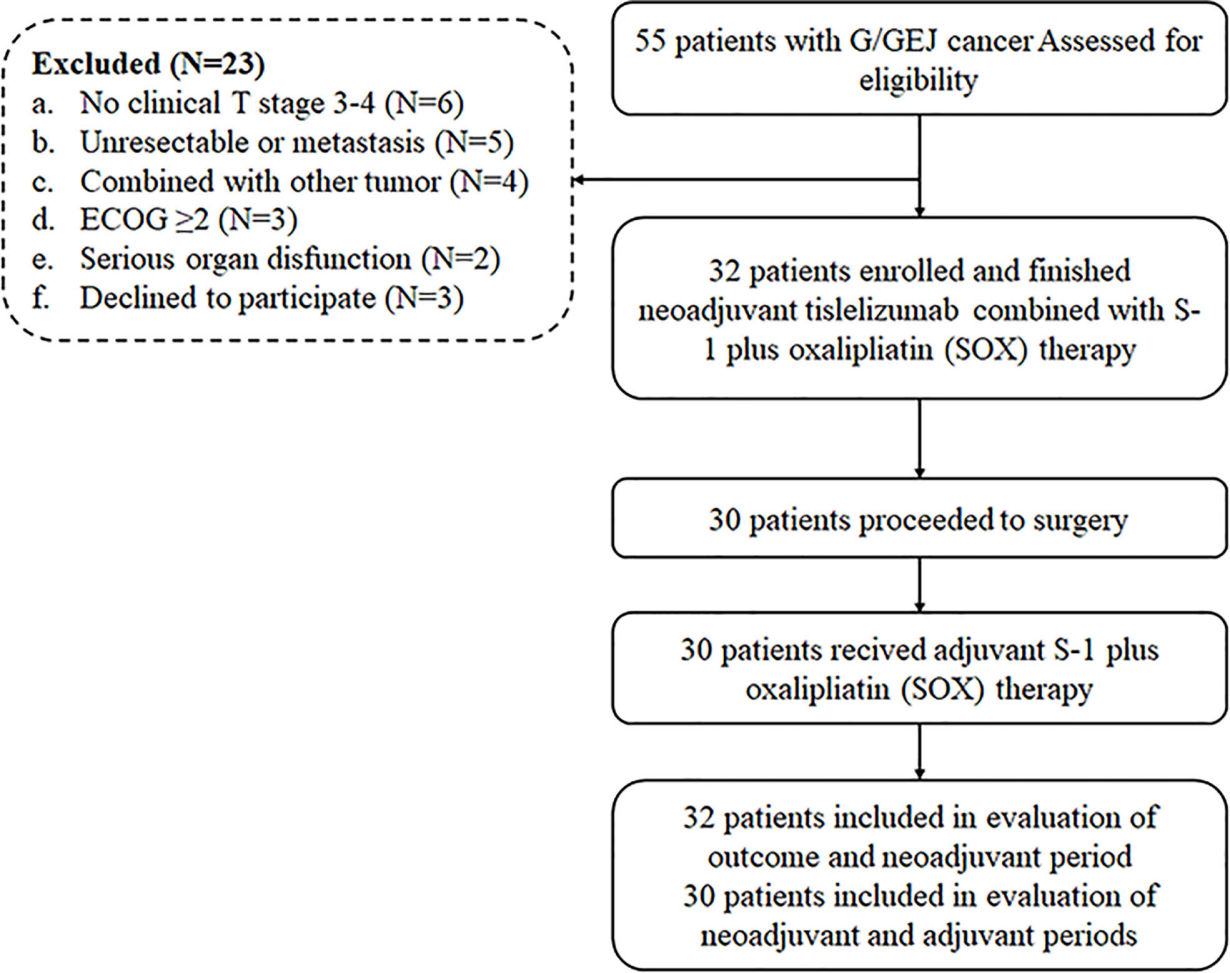
**(A)** The flowchart of the neoadjuvant immunotherapy–chemotherapy scheme.

Thirty-two patients diagnosed as LAG/GEJ cancer from September 2020 to March 2021 were enrolled (**Figure 1**). The median age was 60.5 years (range, 35 to 74), and 27 (84.4%) patients were male. Tumors of 12 (37.5%) patients were located in the gastroesophageal junction. The Borrmann type of cases was mainly type II (50.0%, 16/32). The mean depth of tumor invasion before treatment was 1.68 ± 0.231 cm. There were 17 (53.1%) patients assessed as being at the clinical T4 stage. Detailed clinicopathological characteristics are shown in **Table 1**.

**Table 1:** Baseline clinicopathological characteristics of patients with gastric cancer.

Clinicopathological characteristics	All patients (%)
Age	
Median (range)	60.5 (35, 74)
≤60	16 (50.0)
>60	16 (50.0)
Sex	
Male	27 (84.4)
Female	5 (15.6)
ECOG	
0	26 (81.3)
1	6 (18.7)
Tumor location	
Antrum of stomach	10 (31.2)
Gastric body	7 (21.9)
Fundus of stomach	3 (9.4)
Gastric-esophageal junction	12 (37.5)
Maximal diameter of the tumor (cm)	
≤ 5	6 (18.7)
> 5	26 (81.3)
Depth of tumor invasion before treatment (cm)	
Mean ± SD	1.68 ± 0.231
Borrmann type	
I	4 (12.5)
II	16 (50.0)
III	9 (28.1)
IV	3 (9.4)
cT before treatment (AJCC 8th TNM stage)^a^	
1	0 (0.0)
2	0 (0.0)
3	15 (46.9)
4	17 (53.1)
cN before treatment (AJCC 8th TNM stage)^b^	
0	0 (0.0)
1	20 (62.5)
2	9 (28.1)
3	3 (9.4)
Lauren’s classification	
Diffuse	20 (62.5)
Intestinal	10 (31.2)
Mixed	2 (6.3)
Nerve invasion	
Yes	12 (37.5)
No	20 (62.5)
Vascular invasion	
Yes	7 (21.9)
No	25 (78.1)

All values presented as *n* (%).

^a^Description was evaluated by CT three-dimensional imaging technology.

^b^Assessed by CT three-dimensional imaging technology before treatment.

LAG/GEJ locally advanced gastric/gastroesophageal junction.

### 3.2 Treatment

All patients completed three cycles of the neoadjuvant tislelizumab, S-1 plus oxaliplatin treatment except for one patient who was evaluated as having progressive disease (PD) after two cycles of treatment. There were 30 (93.8%) patients who proceeded to D2 surgery after neoadjuvant treatment. The median interval time between the last dose of tislelizumab and D2 surgery was 28 days (range, 23–49). A total of 25 (83.3%, 25/30) patients received total gastrectomy, and the other 5 (16.7%, 5/30) patients underwent distal gastrectomy. All patients underwent R0 resection. After D2 surgery, all patients received the S-1 and oxaliplatin (SOX) scheme, and the median time interval between surgery and the first dose of adjuvant treatment was 30 days (24–56).

### 3.3 Safety assessment

AEs occurred in 21 (65.6%) of 32 patients in the neoadjuvant schedule, 23 (76.7%) in the postoperative schedule, and 22 (75.9%) of 29 patients in the adjuvant period. Grade III–IV AEs occurred in 4 of 32 patients (12.5%) in the neoadjuvant schedule and 6 of 30 patients (20.7%) in the adjuvant schedule.

During the neoadjuvant scheme, the five most common AEs were leukopenia (*N* = 10, 31.3%), myelosuppression (*N* = 8 25.0%), anemia (*N* = 6, 18.9%), neutrophil count decrease (*N* = 5, 15.6%), and platelet count decrease (*N* = 4, 12.5%). Most AEs were grade I–II, while two (6.3%) patients had a grade III–IV leukopenia and two (6.3%) patients had a grade III–IV myelosuppression and neutrophil count decrease (**Table 2**).

**Table 2:** Treatment-related adverse events for all patients (N=32).

Adverse events	Neoadjuvant schedule (N=32)				Postoperative schedule (N=30)				Adjuvant schedule (N=29)		
	ALL	Grade I-II	Grade III-IV		ALL	Grade I-II	Grade III-IV		ALL	Grade I-II	Grade III-IV
all events, AEs (patients)	21 (65.6)	17 (53.1)	4 (12.5)		23 (76.7)	16 (53.3)	7 (23.3)		22 (75.9)	16 (55.2)	6 (20.7)
Myelosuppression	8 (25.0)	6 (18.9)	2 (6.3)		5 (16.7)	3 (10.0)	2 (6.7)		14 (48.3)	11 (37.9)	3 (10.3)
Leukopenia	10 (31.3)	8 (25.0)	2 (6.3)		7 (23.3)	6 (20.0)	1 (3.3)		11 (37.9)	7 (24.1)	4 (13.8)
Neutrophil count decrease	5 (15.6)	3 (9.4)	2 (6.3)		3 (10.0)	3 (10.0)	0 (0.0)		7 (24.1)	4 (13.8)	3 (10.3)
Platelet count decrease	4 (12.5)	3 (9.4)	1 (3.1)		3 (10.0)	2 (6.7)	1 (3.3)		8 (27.6)	8 (27.6)	0 (0.0)
Hypoproteinemia	2 (6.3)	2 (6.3)	0 (0.0)		5 (15.6)	4 (12.5)	1 (3.3)		7 (24.1)	6 (20.7)	1 (3.4)
Hemophagocytic syndrome	0 (0.0)	0 (0.0)	0 (0.0)		1 (3.3)	0 (0.0)	1 (3.3)		0 (0.0)	0 (0.0)	0 (0.0)
Wound complications	0 (0.0)	0 (0.0)	0 (0.0)		0 (0.0)	0 (0.0)	0 (0.0)		0 (0.0)	0 (0.0)	0 (0.0)
Abdominal infection	0 (0.0)	0 (0.0)	0 (0.0)		3 (10.0)	3 (10.0)	0 (0.0)		0 (0.0)	0 (0.0)	0 (0.0)
Pulmonary infection	0 (0.0)	0 (0.0)	0 (0.0)		5 (15.6)	3 (10.0)	2 (6.7)		0 (0.0)	0 (0.0)	0 (0.0)
Pleural effusion	0 (0.0)	0 (0.0)	0 (0.0)		7 (23.3)	7 (23.3)	0 (0.0)		2 (6.9)	2 (6.9)	0 (0.0)
Emphysema	0 (0.0)	0 (0.0)	0 (0.0)		3 (10.0)	2 (6.7)	1 (3.3)		0 (0.0)	0 (0.0)	0 (0.0)
Arrhythmia	1 (3.1)	1 (3.1)	0 (0.0)		0 (0.0)	0 (0.0)	0 (0.0)		0 (0.0)	0 (0.0)	0 (0.0)
Pulmonary embolism	0 (0.0)	0 (0.0)	0 (0.0)		3 (10.0)	2 (6.7)	1 (3.3)		1 (3.4)	1 (3.4)	0 (0.0)
Dermatitis	5 (15.6)	4 (12.5)	1 (3.1)		0 (0.0)	0 (0.0)	0 (0.0)		4 (13.8)	4 (13.8)	0 (0.0)
Vena thrombosis	0 (0.0)	0 (0.0)	0 (0.0)		2 (6.7)	2 (6.7)	0 (0.0)		1 (3.4)	1 (3.4)	0 (0.0)
Abdominal aortic thrombosis	1 (3.1)	0 (0.0)	1 (3.1)		0 (0.0)	0 (0.0)	0 (0.0)		0 (0.0)	0 (0.0)	0 (0.0)
Gastroparesis	0 (0.0)	0 (0.0)	0 (0.0)		0 (0.0)	0 (0.0)	0 (0.0)		0 (0.0)	0 (0.0)	0 (0.0)
Anemia	6 (18.9)	5 (15.6)	1 (3.1)		13 (43.3)	9 (30.0)	4 (13.3)		8 (27.6)	8 (27.6)	0 (0.0)
Postoperative bleeding	0 (0.0)	0 (0.0)	0 (0.0)		0 (0.0)	0 (0.0)	0 (0.0)		0 (0.0)	0 (0.0)	0 (0.0)
Anastomotic leakage	0 (0.0)	0 (0.0)	0 (0.0)		0 (0.0)	0 (0.0)	0 (0.0)		0 (0.0)	0 (0.0)	0 (0.0)
Ileus	0 (0.0)	0 (0.0)	0 (0.0)		0 (0.0)	0 (0.0)	0 (0.0)		0 (0.0)	0 (0.0)	0 (0.0)
Vomiting	4 (12.5)	4 (12.5)	0 (0.0)		1 (3.3)	1 (3.3)	0 (0.0)		9 (31.0)	7 (24.1)	2 (6.9)
Diarrhea	1 (3.1)	1 (3.1)	0 (0.0)		0 (0.0)	0 (0.0)	0 (0.0)		3 (10.3)	3 (10.3)	0 (0.0)
Constipation	1 (3.1)	1 (3.1)	0 (0.0)		2 (6.7)	2 (6.7)	0 (0.0)		3 (10.3)	3 (10.3)	0 (0.0)
Abdominal distention	0 (0.0)	0 (0.0)	0 (0.0)		0 (0.0)	0 (0.0)	0 (0.0)		0 (0.0)	0 (0.0)	0 (0.0)
Icterus	2 (6.3)	2 (6.3)	0 (0.0)		0 (0.0)	0 (0.0)	0 (0.0)		2 (6.9)	2 (6.9)	0 (0.0)
Acute renal insufficiency (ARI)	0 (0.0)	0 (0.0)	0 (0.0)		1 (3.3)	0 (0.0)	1 (3.3)		0 (0.0)	0 (0.0)	0 (0.0)
Hypohepatia	5 (15.6)	5 (15.6)	0 (0.0)		2 (6.7)	2 (6.7)	0 (0.0)		6 (20.7)	6 (20.7)	0 (0.0)
Gamma-glutamyl transferase increase	3 (9.4)	3 (9.4)	0 (0.0)		2 (6.7)	2 (6.3)	0 (0.0)		7 (24.1)	7 (24.1)	0 (0.0)
Aspartate aminotransferase increase	2 (6.3)	2 (6.3)	0 (0.0)		2 (6.7)	1 (3.3)	1 (3.3)		4 (13.8)	3 (10.3)	1 (3.4)
Alanine aminotransferase increase	2 (6.3)	2 (6.3)	0 (0.0)		2 (6.7)	1 (3.3)	1 (3.3)		5 (17.2)	4 (13.8)	1 (3.4)

All values presented as *n* (%).

All adverse events were defined and evaluated by CTCAE v5.0.

CTCAE the National Cancer Institute Common Terminology Criteria for Adverse Events.

During the postoperative scheme, the five most common AEs were anemia (*N* = 13, 43.3%), leukopenia (*N* = 7, 23.3%), myelosuppression (*N* = 5, 16.7%), hypoproteinemia (*N* = 5, 16.7%), and platelet count and neutrophil count decrease (*N* = 3, 10.0%). Most AEs were grade I–II, while four (13.3%) patients had a grade III–IV anemia, and one (3.3%) patient had a grade III–IV leukopenia, myelosuppression, hypoproteinemia, and platelet count decrease. Unfortunately, one patient died 21 days after surgery due to acute reaction as a result of hemophagocytic syndrome and renal insufficiency.

During the adjuvant scheme, the five most common AEs were myelosuppression (*N* = 14, 48.3%), leukopenia (*N* = 11, 37.9%), vomiting (*N* = 9, 31.0%), platelet count decrease (*N* = 8, 27.6%), and anemia (*N* = 8,27.6%). Most of these AEs were grade I–II, while six (20.7%) patients experienced grade III–IV AEs that included myelosuppression (*N* = 3, 10.3%), leukopenia (*N* = 4, 13.8%), neutrophil count decrease (*N* = 3, 10.3%), vomiting (*N* = 2, 6.9%), and hypoproteinemia (*N* = 1, 3.4%) (**Table 2**).

### 3.4 Tumor response and prognosis outcomes

After the neoadjuvant immunotherapy–chemotherapy scheme was carried out on 28 patients with target lesions and measured by RECIST v1.1, 13 (40.6%) patients were evaluated as having partial response (PR), 12 (37.5%) patients had a stable disease (SD), and 3 (9.4%) patients were assessed to have a progressive disease (PD). The PD patients had liver metastasis and multiple abdominal metastasis, while one of the PD patients was proved to have a pseudo-progression disease after surgery exploration and pathological evaluation. As for the four patients with non-target disease only, they were evaluated as non-CR/non-PD. The overall disease control rate was 90.6%, and the objective response rate was 53.1%.

**Figure 2:**
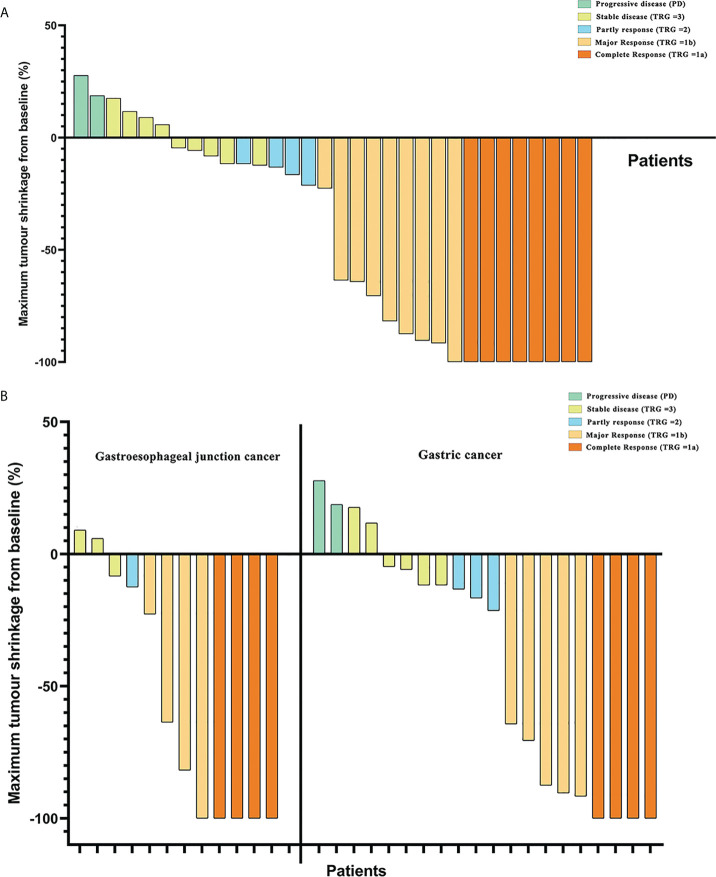
**(A)** Maximum tumor shrinkage in depth of tumor invasion in target lesions from baseline and assessment of tumor responses. **(B)** Comparison of tumor regression grade (TRG) between patients with gastric cancer and gastroesophageal junction cancer in tumor responses.

Based on pathological evaluation, eight (25.0%) patients achieved pCR and were assessed as TRG 1a, and patients with the comparison of imageological and immunohistochemical data pretherapy and post-treatment are shown in **Supplementary Figure S1**. Nine (28.1%) patients had a major response (TRG = 1b) and four patients were assessed as having a partial response (TRG =2), while nine (28.1%) patients were evaluated to have a stable disease (SD) (TRG = 3). The MPR rates of patients with LAG/GEJ cancer were 53.1% (**Table 3**). Comparing MPR rates between gastric cancer and gastroesophageal junction cancer cases showed that there was no significant difference (8/12 vs. 9/20. *χ*^2^ = 1.414, *p* = 0.234) (**Figure 2**).

**Table 3:** Tumor response.

Tumor responses	All patients
**Radiological evaluation**	
RECIST 1.1—no. (%)	*N* = 32 (100)
Patients with target disease	28 (87.5)
Patients with non-target disease only	4 (12.5)
Complete response (CR)	0 (0.0)
Partial response (PR)	13 (40.6)
Stable disease (SD)	12 (37.5)
Progressive disease (PD)	3 (9.4)
True-progression disease	2 (6.3)
Pseudo-progression disease	1 (3.1)
Non-CR/non-PD	4 (12.5)
Objective response	17 (53.1)
Disease control	29 (90.6)
**Pathological evaluation**	
Becker criteria—no. (%)	*N* = 30 (93.8)
Complete response (TRG = 1a)	8 (25.0)
Major response (TRG = 1b)	9 (28.1)
Partial response (TRG = 2)	4 (12.5)
Stable disease (TRG = 3)	9 (28.1)
Major pathology response (TRG = 1a/b)	17 (53.1)

All values presented as n (%).

TRG, tumor regression grade.

**Figure 3:**
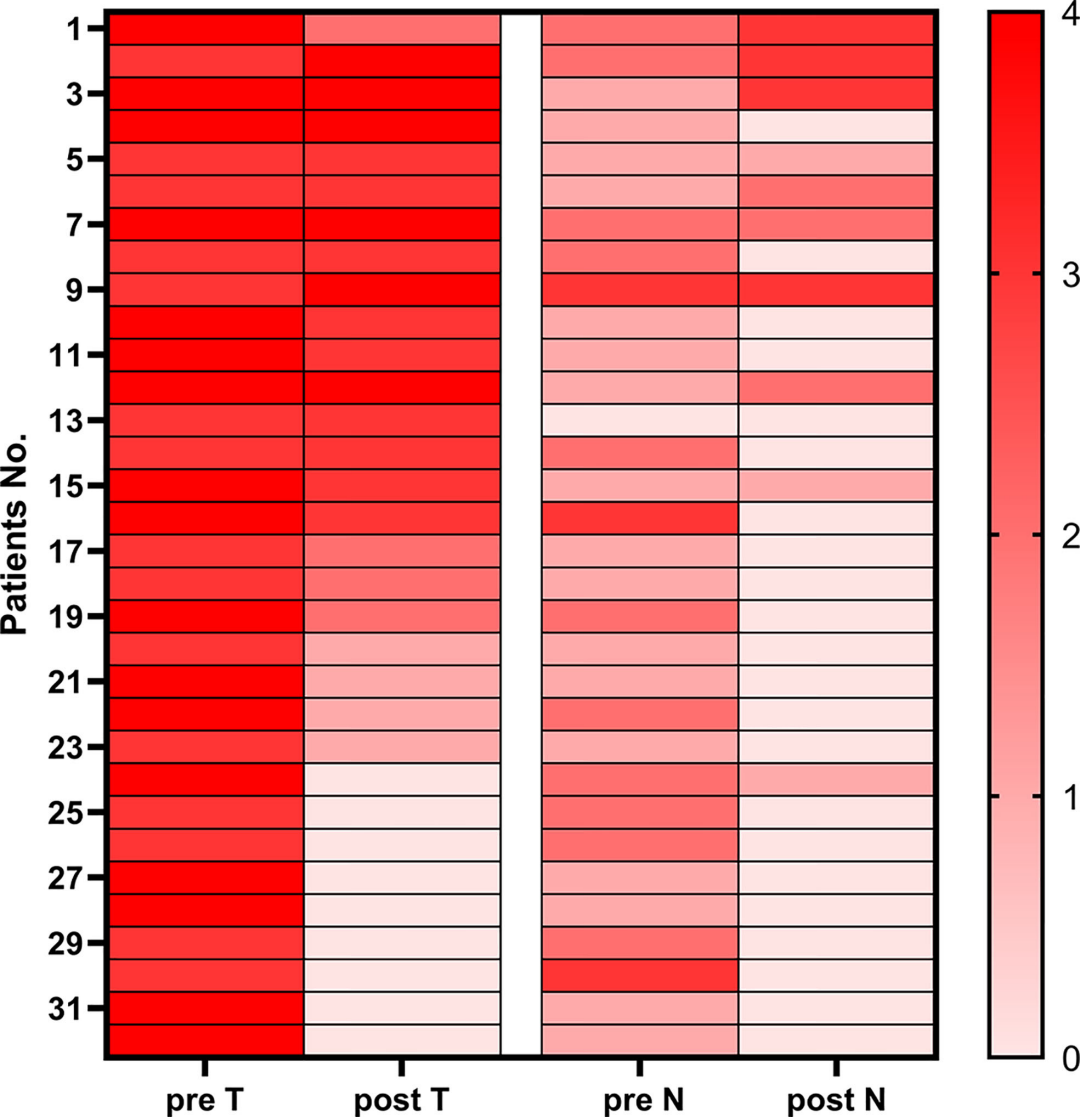
Heatmap of pre-therapy and post-therapy T&N stages of patients with gastric cancer and gastroesophageal junction cancer.

Compared with clinical stage before treatment, there were 21 of 32 (65.6%) patients with LAG/GEJ cancer who achieved degressive T stages and 24 of 32 (75.0%) patients who achieved degressive N stages (**Figure 3**).

**Figure 4:**
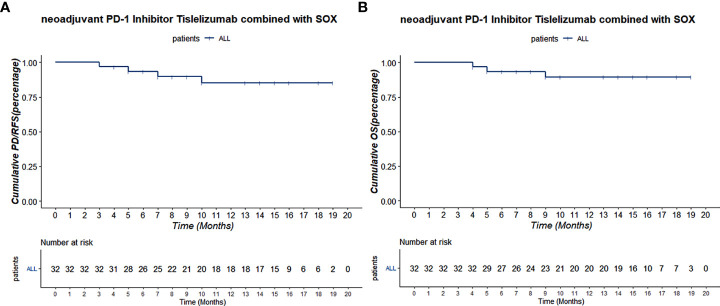
**(A)** The Kaplan–Meier PD/RFS curve of all 32 patients. **(B)** The Kaplan–Meier OS curve of all 32 patients.

The total follow-up time was 19.5 months, and all patients were followed up over 1 year. All patients have been followed till the cutoff date, and the 1-year OS was 91.4%. Two patients were metastasized during the therapy scheme, and one patient died 6 months after laparoscope exploration. The 1-year overall RFS was 90.0%. Median RFS and OS were not reached (**Figure 4**).

### 3.5 Variation of immunological indicators during therapy

We compared the MPR and non-MPR groups, and the results showed significant differences in baseline Hb levels (*Z* = −1.996, *p* = 0.044) and TNFα levels (*Z* = −2.696, *p* = 0.044) (**Supplementary Table S3**). Furthermore, the variations of CD3+ and CD4+ immune cell counts were significantly higher in patients who reached MPR compared with those who did not (CD3+, *p* = 0.044; CD4+, *p* = 0.042). The levels of TNFα in patients who reached MPR was greatly reduced during treatment and TNFα levels were still higher than in patients who did not (*p* = 0.048) (**Supplementary Figure S2**).

### 3.6 Predictive model construction by few-shot learning

We ranked the features and selected TNFα, IFNγ, IL-10, CD4, and age with AUC score > 0.150 through a filtering algorithm (**Supplementary Figure S3A**). The results of correlation analysis showed no significant correlation between those factors (**Supplementary Figure S3B**). Then, we utilized benchmarking operation and results showed that the *k*-nearest neighbor (classif.kknn) classifier had an outstanding performance (classif.ce = 0.268, 95% CI: 0.271–0.354) after resampling (**Supplementary Figure S3C**). The result of the ROC analysis also indicated that the *k*-nearest neighbor (classif.kknn) classifier demonstrated the best accuracy (AUC = 0.824, 95% CI: 0.754–0.899) between classif.kknn, classif.ranger (AUC = 0.684, 95% CI: 0.576–0.787), classif.gausspr (AUC = 0.794, 95% CI: 0.612–0.861), classif. naïve_bayes (AUC = 0.645, 95% CI: 0.513–0.744), classif.svm (AUC = 0.721, 95% CI: 0.611–0.797), classif.fnn (AUC = 0.679, 95% CI: 0.601–0.775), and classif.log_reg (AUC = 0.734, 95% CI: 0.648–0.813) (**Supplementary Figure S3D**).

**Figure 5:**
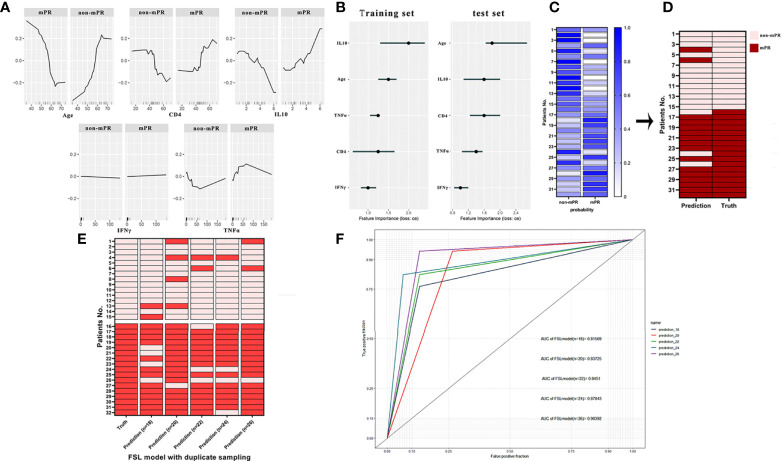
**(A)** The partial dependence plot (PDP) of age, CD4, IFNγ, TNFα, and IL-10 showed the marginal effect features on the predicted mPR outcome of the FSL model. **(B)** The feature importance of age, CD4, IFNγ, TNFα, and IL-10 in the training set and test set. **(C)** Probabilities of predictive outcomes of each patient in the final FSL model. **(D)** Comparison of authentic mPR outcome and predictive outcome of each patient computed by the FSL model. **(E)** Heatmap of actual outcomes and predictive outcomes of the FSL model trained by different training sets. **(F)** ROC analysis of the FSL model trained by different training sets. The receiver operating characteristic curve (ROC).

After that, we chose the *k*-nearest neighbor (classif.kknn) classifier (*k* = 7) as our FSL model and established through several train sets (*n* = 18, 20, 22, 24, and 26) and test set (*n* = 32). In the computation of the feature effect, results explained that there were positive nonlinear correlations between TNFα, IFNγ, IL-10, CD4, age, and MPR outcome while age was a negative factor with a cutoff value of 56 years. Furthermore, the level of IFNγ showed tremendous change in the effect on MPR outcome with a smooth increase (**Figure 5A**). The result of feature importance calculation showed that IL10 and age took a vital component in the FSL model from either the train set or the test set (**Figure 5B**). Then, we constructed the FSL model workflow and figured out the probability of MPR and non-mPR outcome in each patient, as shown in **Figure 5C**. Finally, after selection and several resampling of the FSL model, we obtained a stable model and tested predictive results with an FSL model sensitivity of 93.1% ± 2.175% and an FSL model specificity of 87.5% ± 3.541% (**Figures 5D, E**). The result of ROC analysis in the test set also indicated the favorable predictive ability of the FSL model (AUC = 0.856, 95% CI: 0.823–0.884) (**Figure 5F**).

## 4 Discussion

Our study leveraging preliminary data indicated that SOX regimen combined with the PD-1 inhibitor neoadjuvant scheme followed by laparoscopic D2 radical gastrectomy had considered therapeutic response with a pCR rate of 25% (8/32) and an MPR rate of 53.1% (17/32) and presented with an acceptable safety profile in advanced G/GEJ cancer patients. More importantly, we examined the expression of immune cytokine and a variety of immune cells in enrolled patients; to our knowledge, this is the first study to build a predictive model to evaluate the therapeutic response of chemotherapy combined with immunotherapy.

In the past decades, therapeutic advances such as targeted drugs and ICB inhibitors have improved the outcomes of metastatic gastric cancer patients, but the prognosis of resectable gastric cancer remains poor and the therapy strategy was controversial between Western and Eastern countries. Recently, neoadjuvant therapy was largely prioritized in Eastern countries including China, but the chemotherapy regimen is used in both regions for advanced G/GEJ cancer. The FLOT4 study reported a 16% pCR rate, which was the highest in contrast to other commonly used regimens such as SOX and CapeOx, while the pCR patient was believed to have a better long-term survival in neoadjuvant therapy (22). However, toxicity could not be neglected; 27% of the patients experienced treatment-related serious AEs in the FLOT4 study. Furthermore, another study (23) revealed that the SOX regimen might be a better neoadjuvant regimen than CapeOx, which presented a longer 3-year DFS after D2 gastrectomy (59.4% vs 51.1%). In our study, the SOX regimen combined with the PD-1 inhibitor tislelizumab achieved a further improvement of pCR rate (25%) and MPR rate (53.1%). A previous study reported that another PD-1 inhibitor, sintilimab, combined with CapeOx also achieved a 19.4% pCR rate and a 47.2% MPR rate, indicating that chemotherapy combined with ICB drugs has great application potential in neoadjuvant therapy for advanced G/GEJ cancer patients.

Previous clinical trials (8, 9, 24) demonstrated that the PD-L1 CPS score was a useful marker to predict therapeutic response in ICB monotherapy in many cancer types, and MSI-H/dMMR and EBV infection was correlated with more tumor-infiltrating lymphocytes (TILs) and a higher PD-L1 expression in gastric cancer patients, which indicated that these patients might benefit from immunotherapy (25), but the ratio of MSI-H/dMMR to EBV-positive gastric cancer patients was low (26). Recently, the predictive value of these traditional markers was challenged in the combination therapy of ICB and chemotherapy or radiotherapy in cancer treatment, and there is some controversy on whether ICB combined with chemotherapy could improve the outcome of the cancer patient with low PD-L1 expression. On the one hand, it was reported that adding ICBs to chemotherapy lacked further benefit in first-line therapy of gastric and esophageal adenocarcinoma after analyzing the data of CheckMate-649, KEYNOTE-062, and KEYNOTE-590 and using KMSubtraction (8, 9, 27). On the other hand, the ORIENT-16 trial (ClinicalTrials.gov, NCT03745-170) showed that CapeOX combined with sintilimab improved the prognosis of all unresectable advanced gastric cancer patients in first-line therapy regardless of the expression of PD-L1. Moreover, the rationale 306 study demonstrated a similar finding in which tislelizumab plus chemotherapy also promotes an anticancer effect in locally advanced esophageal squamous cell cancer. In our single-arm study, the initial enrolled patients were not enough and we need to further expand the number of enrolled patients to determine whether the PD-1 CPS score was a key factor to influence the therapeutic response to neoadjuvant therapy in advanced gastric cancer.

RECIST v1.1 was commonly applied by clinicians to radiologically evaluate the tumor response to neoadjuvant therapy in a series of solid malignant diseases, but its validity was questioned in gastric cancer. In our study, three patients were evaluated to have disease progression by CT scan, one of whom had significant symptom improvement. Thus, we performed laparoscopic exploration and radical gastrectomy, and pathologic examination proved pCR in this case; moreover, no patient was evaluated to have MPR or cCR by preoperative radiological examination in patients who achieved MPR or pCR. Thus, we developed a novel model to predict the therapeutic response *via* the data of immune-related cytokines and immune cell proportion during neoadjuvant therapy. Immune cytokines modulate the adaptive and innate response in the tumor microenvironment, and the anticancer activity was validated in many preclinical models. We also observed significant elevation of some cytokines as well as changes in the proportion of immune cells in MPR patients but not in non-MPR patients; this inspired us to determine whether such variety could create a model to predict the response to our scheme, and the CD3^+^, CD4^+^, and TNFα levels composed the FSL model, which achieved a gratifying predictive role. To our knowledge, this is the first model to recognize the disadvantage of radiologic evaluation of neoadjuvant therapy in gastric cancer.

The most frequent AE of this study was leukopenia, and 10 (31.3%) patients experienced it during the neoadjuvant period. Nevertheless, the frequency of leukopenia was less than that of neoadjuvant ECF/ECX and FLOT formula reported in the FLOT4 study (22). Meanwhile, anemia and myelosuppression were the second most common AEs in this study, which presented with similar frequency as reported in a previous neoadjuvant chemotherapy study (28). The frequency of rash or dermatitis was 5 (15.6%) in this study, which was consistent with historical data of tislelizumab (29). Satisfactorily, only four (12.5%) patients developed grade III–IV AEs; unfortunately, one patient died during the postoperative period due to hemophagocytic syndrome, which was recognized as the most serious AE during immunotherapy; this reminded us to pay more attention to and improve management during immunotherapy. On the whole, the majority of ocular immune-related adverse events (irAEs) are mild and low-grade, which might be attributed to the cycle of our study being short.

Despite the novel findings, there were some limitations in this study. Firstly, although a previous retrospective study demonstrated that advanced gastric cancer patients who achieved pCR had a better clinical outcome than non-pCR patients after adjuvant chemotherapy, the follow-up time of our study was short; thus, we need to prolong the follow-up time to determine whether adding tislelizumab to the neoadjuvant SOX regimen elevated the prognosis of advanced G/GEJ patients. Secondly, we need to increase the number of enrolled patients to further optimize our predictive model. Thirdly, once the number of patients is enough, traditional predictive markers should be systematically examined, such as PD-L1 CPS score, MMR status, and EBV infection.

In conclusion, the neoadjuvant PD-1 inhibitor tislelizumab combined with chemotherapy has promising therapeutic application potential in patients with advanced G/GEJ cancer and presented an acceptable safety profile. Meanwhile, CD3^+^, CD4^+^, and TNFα levels might be valuable immune indicators of tumor response, and should be paid more clinical attention. Based on our findings, we developed a predictive model that may be helpful for precision medication and personalized treatment for advanced G/GEJ patients.

## Data availability statement

The data that support the findings of this study are available from the corresponding author, KT, upon reasonable request. Requests to access the datasets should be directed to KT, kaixiongtao@hust.edu.cn.

## Ethics statement

The studies involving human participants were reviewed and approved by Human Ethics Committee of Union Hospital, Tongji Medical College, Huazhong University of Science and Technology. The patients/participants provided their written informed consent to participate in this study. Written informed consent was obtained from the individual(s) for the publication of any potentially identifiable images or data included in this article.

## Author contributions

KT and PZ were in charge of the study concepts and design. YY and YL executed the clinical trial and drafted the manuscript. JBL, JYL, and MY controlled the quality of data. KW, AL, and KL evaluated the endpoints. KC, XS, ZW, and JS revised the manuscript. All authors read approved the final manuscript.

## Funding

This study was funded by the National Natural Science Foundation of China (Nos. 82003205, 81702386, and 81874184) and Hubei Provincial Department of Science and Technology (No.2021BCA116).

## Acknowledgments

We are grateful to the pathologists working in Wuhan Union Hospital for their help in the assessment of tumor responses and in conducting this study.

## Conflict of interest

The authors declare that the research was conducted in the absence of any commercial or financial relationships that could be construed as a potential conflict of interest.

The reviewer CY declared a shared parent affiliation with the author JS to the handling editor at the time of review.

## Publisher’s note

All claims expressed in this article are solely those of the authors and do not necessarily represent those of their affiliated organizations, or those of the publisher, the editors and the reviewers. Any product that may be evaluated in this article, or claim that may be made by its manufacturer, is not guaranteed or endorsed by the publisher.

## Supplementary material

Supplementary Figure 1 Comparation of **(A)** high resolution computerized tomography (CT), **(B)** hematoxylin-eosin staining in lesions, **(C)** esophagogastroscopy results between patients pretherapy and after all cycles of treatment.

Supplementary Figure 2 Variation of immunity indicators during cycles of treatment.

Supplementary Figure 3 **(A)** A rank of the features based on the AUC value to the response calculated by filtering algorithm; **(B)** Correlation analysis of selected features age, CD4, IFNγ, TNFα and IL-10. **(C)** comparation analysis of the classification error of six FSL machine-learning classifiers. **(D)** ROC analysis of six FSL machine-learning classifiers. The area under the ROC curve AUC, the receiver operating characteristic curve ROC.
