# Supplementary figures and images for: Neoadjuvant tislelizumab and tegafur/gimeracil/octeracil (S-1) plus oxaliplatin in patients with locally advanced gastric or gastroesophageal junction cancer: Early results of a phase 2, single-arm trial
Source: Front Oncol. 2022 Aug 30;12:959295. doi: 10.3389/fonc.2022.959295 (PMC9491115; doi:10.3389/fonc.2022.959295)

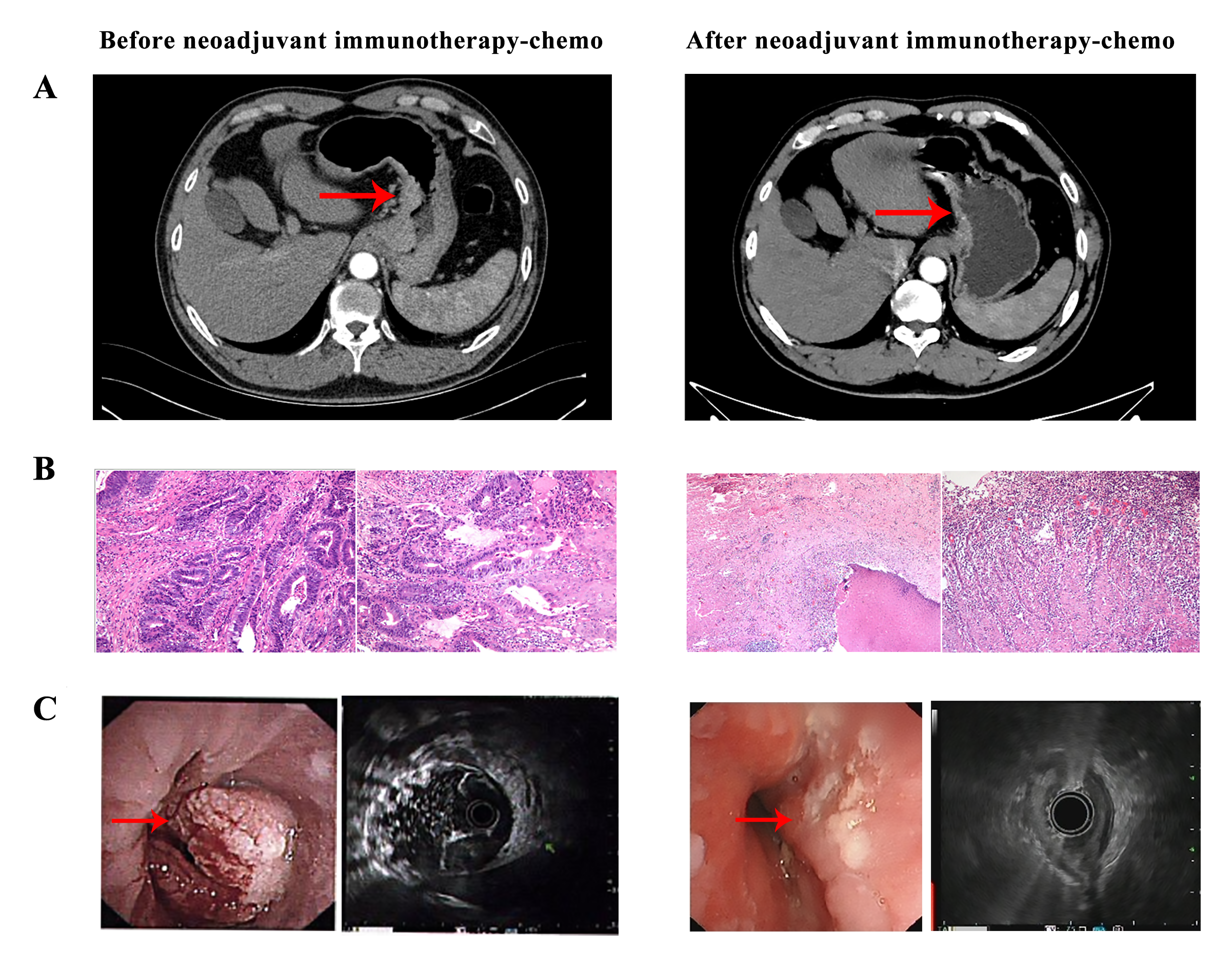

Supplement: Supplementary Figure 1 — Comparation of (A) high resolution computerized tomography (CT), (B) hematoxylin-eosin staining in lesions, (C) esophagogastroscopy results between patients pretherapy and after all cycles of treatment. [file Image_1.tif]

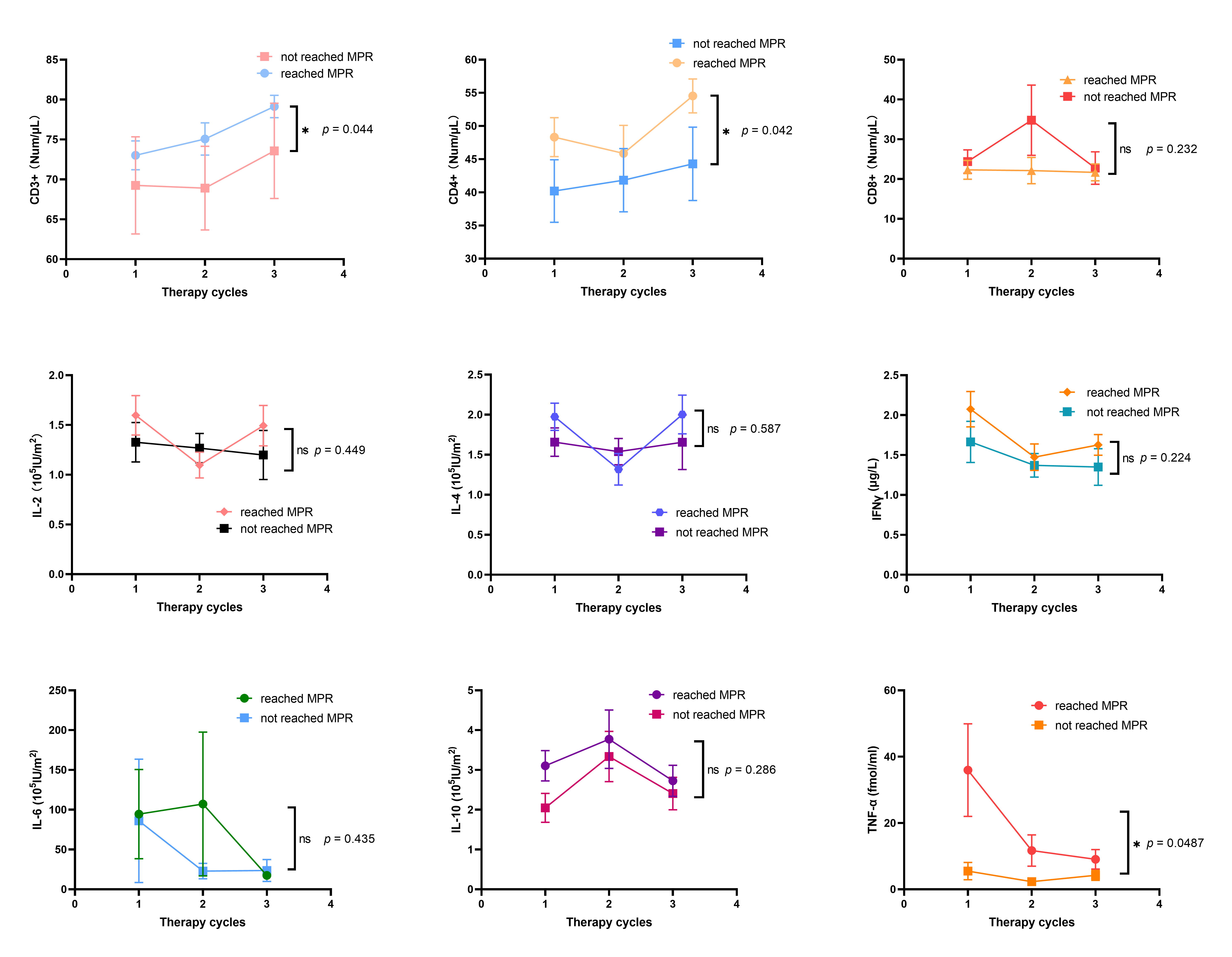

Supplement: Supplementary Figure 2 — Variation of immunity indicators during cycles of treatment. [file Image_2.tif]

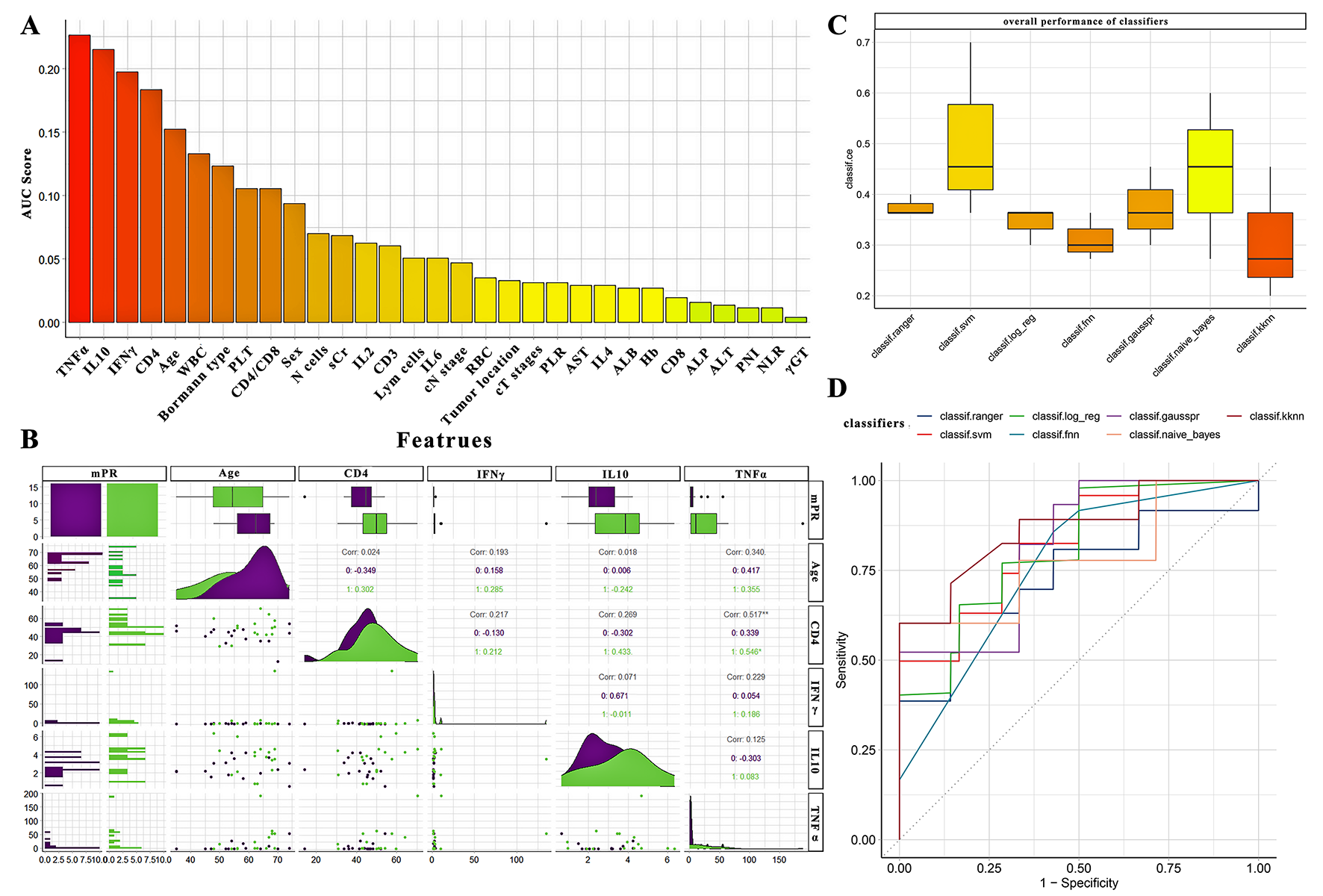

Supplement: Supplementary Figure 3 — (A) A rank of the features based on the AUC value to the response calculated by filtering algorithm; (B) Correlation analysis of selected features age, CD4, IFNγ, TNFα and IL-10. (C) comparation analysis of the classification error of six FSL machine-learning classifiers. (D) ROC analysis of six FSL machine-learning classifiers. The area under the ROC curve AUC, the receiver operating characteristic curve ROC. [file Image_3.tif]
