# Supplementary material for: Neoadjuvant tislelizumab and tegafur/gimeracil/octeracil (S-1) plus oxaliplatin in patients with locally advanced gastric or gastroesophageal junction cancer: Early results of a phase 2, single-arm trial
Source: Front Oncol. 2022 Aug 30;12:959295. doi: 10.3389/fonc.2022.959295 (PMC9491115; doi:10.3389/fonc.2022.959295)
Supplement: Supplementary file 4 [file Table_1.docx]

**Supplementary Table S1 Eligibility**

| **Minimum Age: 18 Years; Maximum Age: 75 Years; Sex: All** | |
| --- | --- |
| **Inclusion Criteria** | **exclusion Criteria** |
| 1. Obtaining written informed consent from patient in advance; 2. Gastroscopy confirmed adenocarcinoma of the gastroesophageal junction or stomach and biopsy pathology confirmed adenocarcinoma of the stomach; 3. ECOG 0-1 4. Normal function of major organs, meeting the following criteria: 5. Blood routine test criteria should be met (Patients for 14 days were not transfused with blood products and not corrected with G-CSF and other hematopoietic stimulating factors)    1. HB≥90 g/L；    2. ANC≥1.5×10^9/L；    3. PLT≥125×10^9/L； 6. Chemistry panel meeting the following criteria:    1. TBIL< 1.5ULN;    2. ALT and AST< 2.5ULN, but< 5ULN for patients with liver metastasis;    3. serum Cr ≤ 1.25ULN or endogenous creatinine clearance > 50ml/min (Cockcroft-Gault formula); 7. Women of childbearing potential must have used reliable contraception or had a pregnancy test (serum or urine) within 7 days prior to enrollment and had a negative result, and be willing to use an appropriate method of contraception during the trial and for 8 weeks after administration of the trial drug. For men, agree to use an appropriate method of contraception or have been surgically sterilized during the trial and for 8 weeks after receiving study drug; 8. Advanced gastric cancer as assessed by ultrasonography and/or gastric CT (cT3-T4a, N+, M0). | 1. Peritoneal dissemination; 2. Patients who previously received platinum, fluoropyrimidine chemotherapy or targeted therapy, and patients who received radiotherapy to target lesion during combined therapy; 3. Multiple factors affecting oral medications (e.g., inability to swallow, chronic diarrhea, bowel obstruction, etc.); 4. History of melena or hematemesis in the past three months, or patients with high-risk bleeding such as intestinal perforation, gastric perforation, large area ulcer, or patients with active digestive ulcer lesions in the stomach and fecal occult blood (+ +); 5. Patients with hypertension and uncontrolled by antihypertensive treatment alone (systolic blood pressure>140 mmHg, diastolic blood pressure>90 mmHg); patients with a history of unstable angina; patients newly diagnosed as angina pectoris within 3 months before screening or myocardial infarction events within 6 months before screening; arrhythmia (including QT ≥ 450 ms for men, ≥ 470 ms for women) requiring long-term use of antiarrhythmic drugs and New York Heart Association class ≥ II cardiac insufficiency; Doppler ultrasound assessment: ejection fraction (LVEF) < 50%; 6. Urine routine showed urine protein ≥ (++) and confirmed 24-hour urine protein >1.0g; 7. Long-term unhealed wounds or incompletely healed fractures; 8. Patients with abnormal coagulation function and bleeding tendency (the following criteria must be met within 14 days before enrollment: INR is within normal range without anticoagulant); patients treated with anticoagulants or vitamin K antagonists such as warfarin, heparin or their analogues; patients treated with low-dose warfarin (1 mg orally, once daily) or low-dose aspirin (the daily dose does not exceed 100 mg) for preventive purposes are allowed under the premise of prothrombin time international normalized ratio (INR) ≤ 1.5; 9. Hyperactive/venous thrombotic events within 1 year before screening, such as cerebrovascular accident (including transient ischemic attack), deep venous thrombosis (except venous thrombosis caused by previous chemotherapy that has been cured by the investigator) and pulmonary embolism; 10. Patients with a history of psychotropic substance abuse who cannot quit or have mental disorders; 11. Patients with concomitant diseases that seriously jeopardize the patient's safety or affect the patient's completion of the study as judged by the investigator; 12. Pregnant or lactating women. |

**Supplementary Table S2 Arms and Interventions**

| Arms | Assigned Interventions |
| --- | --- |
| Experimental: PD-1 with SOX  S-1: 40~60mg Bid, d1~14, q3w Oxaliplatin: 130mg/m2, iv drip for 2h, d1, q3w PD-1 (Tislelizumab):200mg, iv drip for at least 1h, d1, q3w | Drug: PD-1 inhibitor (Tislelizumab), SOX (S-1+ Oxaliplatin)  S-1: 40~60mg Bid, d1~14, q3w. Oxaliplatin: 130mg/m2, iv drip for 2h, d1, q3w. PD-1 (Tislelizumab) :200mg, iv drip for at least 1h, d1, q3w. Stage I: PD1 inhibitor + SOX regimen (q3W). Stage II: PD1 inhibitor + SOX regimen (q3W) Stage III: PD1 inhibitor + SOX regimen (q3W). Efficacy evaluation, then followed by surgery |

| **Supplementary Table S3. Comparation of hematological indicators of GC patients before treatment** | | | | | |
| --- | --- | --- | --- | --- | --- |
|  | All patients | MPR | non-MPR | Z value | *p* value |
| Routine hematological indicators | | | | | |
| WBC (◊10^9^) | 5.71 ± 2.01 | 6.27 ± 2.45 | 5.15 ± 1.29 | -1.282 | 0.200 |
| RBC (◊10^12^) | 3.91 ± 0.57 | 3.89 ± 0.56 | 3.94 ± 0.60 | -0.339 | 0.734 |
| PLT (◊10^9^) | 230.44 ± 71.26 | 238.50 ± 51.85 | 222.38 ± 87.56 | -1.018 | 0.309 |
| Hb (g/L) | 113.66 ± 21.37 | 113.81 ± 21.02 | 113.50 ± 22.41 | -0.264 | 0.792 |
| N (◊10^9^) | 3.77 ± 1.76 | 4.13 ± 2.17 | 3.41 ± 1.19 | -0.528 | 0.598 |
| Lym (◊10^9^) | 1.28 ± 0.57 | 1.34 ± 0.61 | 1.22 ± 0.55 | -0.678 | 0.497 |
| PLR | 212.56 ± 112.01 | 225.59 ± 148.13 | 200.26 ± 62.22 | -0.302 | 0.763 |
| NLR | 3.57 ± 1.54 | 3.86 ± 1.22 | 3.44 ± 1.95 | -0.113 | 0.910 |
| ALP (U/L) | 65.16 ± 16.90 | 65.19 ± 16.61 | 65.13± 17.72 | -0.151 | 0.880 |
| ALT (U/L) | 15.44 ± 7.66 | 14.88 ± 5.76 | 16.00 ± 9.35 | -0.133 | 0.894 |
| AST (U/L) | 20.63 ± 11.20 | 20.81 ± 12.30 | 20.44 ± 10.39 | -0.285 | 0.776 |
| γGT (U/L) | 18.97 ± 12.56 | 17.25 ± 6.16 | 20.69 ± 16.78 | -0.038 | 0.970 |
| ALB (g/L) | 37.42 ± 4.02 | 37.23 ± 4.03 | 37.60 ± 4.14 | -0.264 | 0.792 |
| PNI | 43.81 ± 5.27 | 43.94 ± 4.76 | 43.68 ± 5.90 | -0.113 | 0.910 |
| sCr (μmol/L) | 76.41 ± 16.56 | 75.606 ± 19.24 | 77.22 ± 13.97 | -0.660 | 0.509 |
| Immunological indicators | | | | | |
| CD3 (%) | 70.51 ± 12.23 | 72.03 ± 12.33 | 68.99 ± 12.34 | -0.584 | 0.559 |
| CD4 (%) | 44.73 ± 11.05 | 47.50 ± 12.14 | 41.96 ± 9.41 | -1.139 | 0.187 |
| CD8 (%) | 22.29 ± 8.86 | 21.80 ± 8.95 | 22.78 ± 9.04 | -0.189 | 0.850 |
| IL2 (μg/L) | 3.71 ± 10.88 | 5.49 ± 15.40 | 1.93 ± 0.77 | -0.603 | 0.546 |
| IL4 (μg/L) | 1.88 ± 0.64 | 1.89 ± 0.65 | 1.86 ± 0.64 | -0.283 | 0.777 |
| IL6 (μg/L) | 43.39 ± 133.33 | 76.66 ± 185.15 | 10.12 ± 9.81 | -0.490 | 0.624 |
| IL10 (μg/L) | 3.21 ± 1.48 | 2.67 ± 1.02 | 3.76 ± 1.68 | -2.149 | 0.032 |
| TNFα (pg/ml) | 19.93 ± 36.46 | 31.09 ± 47.58 | 8.78 ± 14.79 | -2.187 | 0.029 |
| IFNγ (μg/L) | 6.04 ± 23.53 | 10.21 ± 33.26 | 1.86 ± 0.67 | -0.697 | 0.486 |

All values presented as *Mean* ± *SD*

Comparisons between hematological indicators of patients with MPR and non-MPR were made by the Mann-Whitney U rank sum test.

PLR, platelet-lymphocyte ratio; NLR, neutrophil-lymphocyte ratio; γGT, γ-glutamyl transpeptidase; PNI, prognostic nutritional index; sCr, serum creatinine; TNFα, tumor necrosis factor α; IFNγ, interferon γ.
